# Updating Understanding of Macular Microvascular Abnormalities and Their Correlations With the Characteristics and Progression of Macular Edema or Exudation in Coats' Disease

**DOI:** 10.3389/fmed.2022.788001

**Published:** 2022-04-13

**Authors:** Juan Zhang, Lu Ruan, Chen Jiang, Qian Yang, Yuqiao Ju, Qing Chang, Xin Huang

**Affiliations:** ^1^Eye and ENT Hospital, Shanghai Medical College, Fudan University, Shanghai, China; ^2^Shanghai Key Laboratory of Visual Impairment and Restoration, Fudan University, Shanghai, China

**Keywords:** Coats' disease, macular microvascular abnormalities, macular edema, macular exudation, multimodal imaging, optical coherence tomography angiography

## Abstract

**Objective:**

To investigate the associations of macular microvascular abnormalities with the characteristics and progression of macular edema or exudation in Coats' disease, toward an updated understanding of possible risk factors for macular edema or exudation.

**Methods:**

Twenty-six eyes (26 patients) with Coats' disease and macular edema or exudation underwent multimodal imaging and were followed for 18 months. The eyes were classified according to their outcomes (refractory or improved). Macular capillary affections were assessed by optical coherence tomography angiography (OCTA) and fluorescein angiography (FA). Histopathological analysis of the macular region of an additional enucleated eye was performed.

**Results:**

OCTA revealed telangiectasia in the deep capillary plexus (DCP) in 76.9% and the superficial capillary plexus (SCP) in 34.6% of 26 eyes with macular edema or exudation of Coats' disease, exceeding the rate detected by FA (21.4%). Eyes with intraretinal cystoid spaces/exudates of the macula presented higher presence of telangiectasia in the SCP (57.1% with vs. 8.3% without, *X*2 = 6.801, *P* = 0.009) and DCP (92.9 with vs. 58.3% without, *X*2 = 4.338, *P* = 0.037). The parafoveal vessel densities (VDs) and fractal dimension in the SCP and DCP were lower in affected eyes than in contralateral eyes (all *P* < 0.001). The VD in SCP (*P* = 0.009) and DCP (*P* = 0.010) were lower in refractory group than in improved group. Dilated capillaries with incomplete vessel walls and adjacent inflammatory cells were detected in the neuroretina of the macula in histopathological specimen.

**Conclusions:**

Macular capillary abnormalities, including telangiectasia and VD loss, were positively detected in eyes with macular edema or exudation of Coats' disease. Intraretinal cystoid spaces/exudates of the macula, rather than subretinal exudates, may be related to macular telangiectasia. VD losses in the SCP and DCP may be risk factors for refractory macular edema or exudation.

## Introduction

Coats' disease is a rare idiopathic disorder that is unilateral in >75% of patients and predominantly affects pediatric males (1–3). Distinctive features of this disease include retinal vascular abnormalities and massive retinal exudation (2, 4). The retinal vascular abnormalities, usually described as telangiectasias or microaneurysms, present as dilation or tortuosity of the capillary network and cause progressive accumulation of lipid exudates (5–7).

Earlier in this century, Shields and his colleagues summarized the clinical characteristics of Coats' disease and proposed a classification system. These now guide our understanding of Coats' disease, including its diagnosis and management (2). As reported, exudation or edema are common in the macular region in eyes with Coats' disease, resulting in severe visual loss, however, the causes of macular edema or exudation have been largely unknown. The presence of retinal vascular abnormalities in Coats' disease, detected by fundus photography and fluorescein angiography (FA), was located predominantly in peripheral regions of the temporal and lower quadrants of the retina, but was rare in the macular region (2–4, 8). Considering this discrepancy, it was hypothesized that macular exudation could be caused by peripheral retinal vascular abnormalities (2). However, it is difficult to obtain highly detailed images of the microvasculature using FA, especially for the deep capillary plexus (DCP) (9, 10). This may imply that microvascular abnormalities in the macular region of Coats' disease may be undetected due to imaging limitations. Therefore, the existence of microvascular abnormalities in the macular region is still inconsistent and confusing.

Advanced imaging techniques are therefore needed to provide more understanding of the macular microvascular features and their relationship with the characteristics and progression of macular edema or exudation in Coats' disease. Optical coherence tomography angiography (OCTA) is a non-invasive imaging modality that can be used to visualize and quantify blood flow in each retinal capillary layer. In recent years, telangiectasia and capillary dropout in the macular region of eyes with Coats' disease were detected using OCTA in some case series and studies with small numbers of patients (8, 11–13). Those findings remind us that macular microvascular abnormalities may occur in eyes affected by Coats' disease, but the clinical significance of these abnormalities, especially their associations with macular edema or exudation, are still unknown. Besides, macular edema or exudation presenting refractory progression is also a great challenge to clinical treatment of Coats' disease and cause severe visual loss, but studies on its risk factors have been almost margin.

In consideration of these apparent confusions, we conducted multimodal imaging analysis of a relatively large sample of patients with Coats' disease in order to obtain qualitative and quantitative information about the presence of macular microvascular abnormalities in eyes with macular edema or exudation. Further, we examined the associations between the macular microvascular abnormalities with the classification and progression of macular edema or exudation cross-sectionally and longitudinally. In addition, we combined histopathological analysis to propose more evidence for this relationship. Together, we hope to expand our understanding of the associations of macular vascular abnormalities with macular edema or exudation in Coats' disease.

## Materials and Methods

### Study Population and Classification

This retrospective cohort study included consecutive patients (aged <21 years) who were diagnosed with Coats' disease at the Shanghai Eye and ENT Hospital of Fudan University from January 1, 2017, to February 28, 2020. Patients diagnosed with unilateral Coats' disease exhibiting macular exudation or edema were eligible if quantitative OCTA images at baseline and over a long follow-up of over 18 months were available. Affected eyes with a history of ocular therapy at baseline, retinal detachment involving the macula, or obvious media opacities were excluded, as were contralateral eyes with visible telangiectasia or exudates. Patients who were followed for less than 18 months and patients with a history of other systemic or ocular diseases were also excluded.

Widefield fundus photography (Optos 200TX, Dunfermline, Scotland, UK) and slit lamp examination were conducted in both eyes of all patients for diagnosis and clinical staging of Coats' disease. The diagnostic criteria for Coats' disease were the presence of an idiopathic ocular disorder manifested as retinal telangiectasia together with intraretinal or subretinal exudation. Coats' disease was staged according to the criteria proposed by Shields et al. (8, 11–13). For all contralateral eyes, widefield fundus photography were conducted at each follow-up visit, no vascular abnormalities and exudates were detected in these contralateral eyes during the whole follow-up for over 18 months, ensuring that these contralateral eyes were disease-free. Other data, including best-corrected visual acuity (BCVA) (logarithms of the minimum angle of resolution [logMAR]) at baseline and after follow-up detected via standard logarithmic visual acuity chart, axial length (AL) detected via A-mode ultrasound, and demographic characteristics were recorded.

At baseline, macular lesions were divided into macular edema or exudation based on the findings of spectral-domain optical coherence tomography (SD-OCT; Heidelberg Engineering, Heidelberg, Germany) and widefield fundus photography. Macular edema presented as intraretinal hyporeflective cystoid spaces on SD-OCT images. Intraretinal and subretinal hyperreflective exudates, presenting in the macular region on SD-OCT images, were collectively expressed as macular exudation, which corresponded with yellow exudates on widefield fundus photography (7). Concomitant macular edema was defined as hyporeflective intraretinal cystoid spaces combined with hyperreflective exudates in the macular region. To analyze the association of location of macular lesions (cystoid or exudates) in different retinal layers with macular microvascular abnormalities, macular lesions were divided as intraretinal lesions and subretinal lesions, respectively, intraretinal lesions in the macular region were defined as cystoid or exudates involving the neuroretina, whereas subretinal lesions were defined as exudates or fluid located only under the neuroretina.

The study was performed in accordance with the tenets of the Declaration of Helsinki and was approved by the Ethics Review Board of the Shanghai Eye and ENT Hospital of Fudan University. The patients or their parents provided signed informed consent.

### Macular Microvascular Analysis

At baseline, each patient underwent binocular OCTA using the AngioVue OCTA system (Optovue Inc., Fremont, CA) with the commercial built-in software (Avanti RTVue-XR, version 2017.1.0.155). A scan area of 3 × 3 mm, centered on the fovea, was acquired to detect vessel abnormalities in the macular region. Patients were instructed to immobilize the internal target. The initial alignment, lighting and focus of the camera was done in infrared (IR) mode with the help of the machine's “auto all” feature. OCTA images were obtained by two consecutive scans of orthogonal registration and merging. Image quality review was conducted for all scans, signal strength index (SSI) was used to quantitatively measure the quality of images. Simultaneous widefield FA at the first visit was conducted for both eyes in 14 enrolled patients to assist the vascular analysis.

The macular region was defined as a circle extending from the foveola with a radius of 3.0 mm. The superficial capillary plexus (SCP) en face image was sectionalized from the internal limiting membrane to 15 μm above the inner plexiform layer (IPL), while the deep capillary plexus (DCP) en face image was segmented from 15 μm above the IPL to 10 μm below the outer plexiform layer. The SCP and DCP were automatically segmented by the software and manually adjusted if the automatic segmentation was inaccurate. The parafoveal vessel densities (VDs) of the SCP and DCP were measured using the density function of the built-in software. The retinal thickness of the whole foveal and parafoveal layer were also measured using this system. The unqualified images with low SSI (<8/10), severe segmentation errors that could not be adjusted manually and artifacts resulting from poor clarity, blink fixation and poor fixation were excluded for analysis.

**Figure 1:**
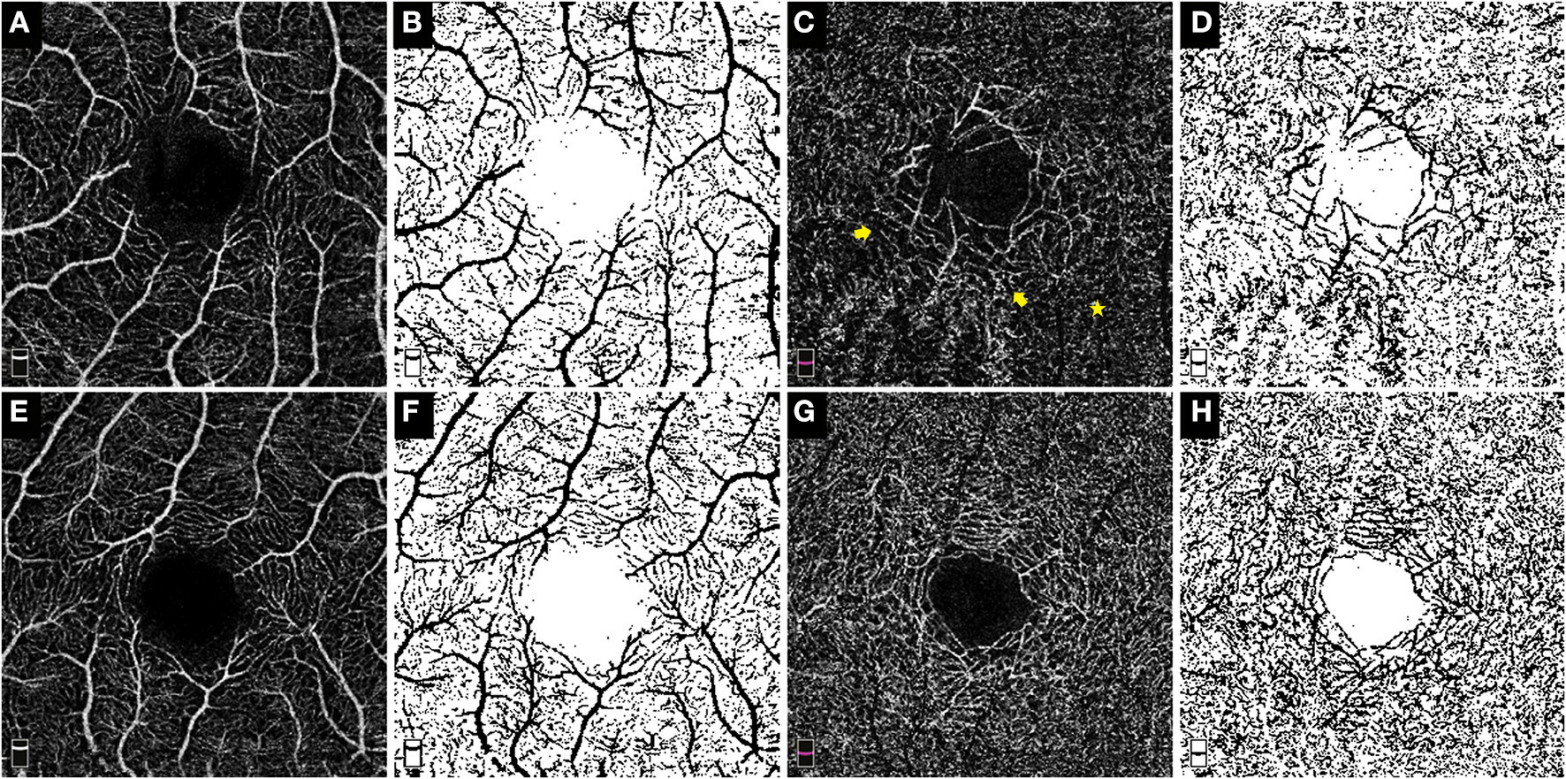
OCTA enface images and the corresponding post-processed, standardized and binarized images. The OCTA enface images (3 × 3 mm) of the SCP **(A)** and DCP **(C)** in eye with Coats' disease and their binarized images in both plexuses **(B,D)** respectively. The OCTA enface images of the SCP **(E)** and DCP **(G)** in the contralateral eye and their binarized images in both plexuses [**(F,H)**, respectively]. No obvious dilated and tortuous capillaries structures were detected in the SCP of this 10-year-old boy **(A)**; in the DCP, the capillaries around the foveal avascular zone were obviously more dilated (the yellow arrow) in contrast to the surrounding capillaries (the yellow star), therefore, presence of microvascular abnormalities in the DCP of this eye was positive.

The fractal dimension (FD), a widely used parameter to present fractal analysis, has been used to measure the architecture of the retinal vasculature determined by FA and OCTA (14–16). The FD was determined using the “box-counting” technique in Fractalyse software (ThéMA, Besancon Cedex, France) after the OCTA images had been standardized and binarized using ImageJ software (National Institutes of Health, Bethesda, MD), as described in previous studies (17). Figure 1 shows representative OCTA en face images of SCP and DCP and the corresponding standardized and binarized images.

**Figure 2:**
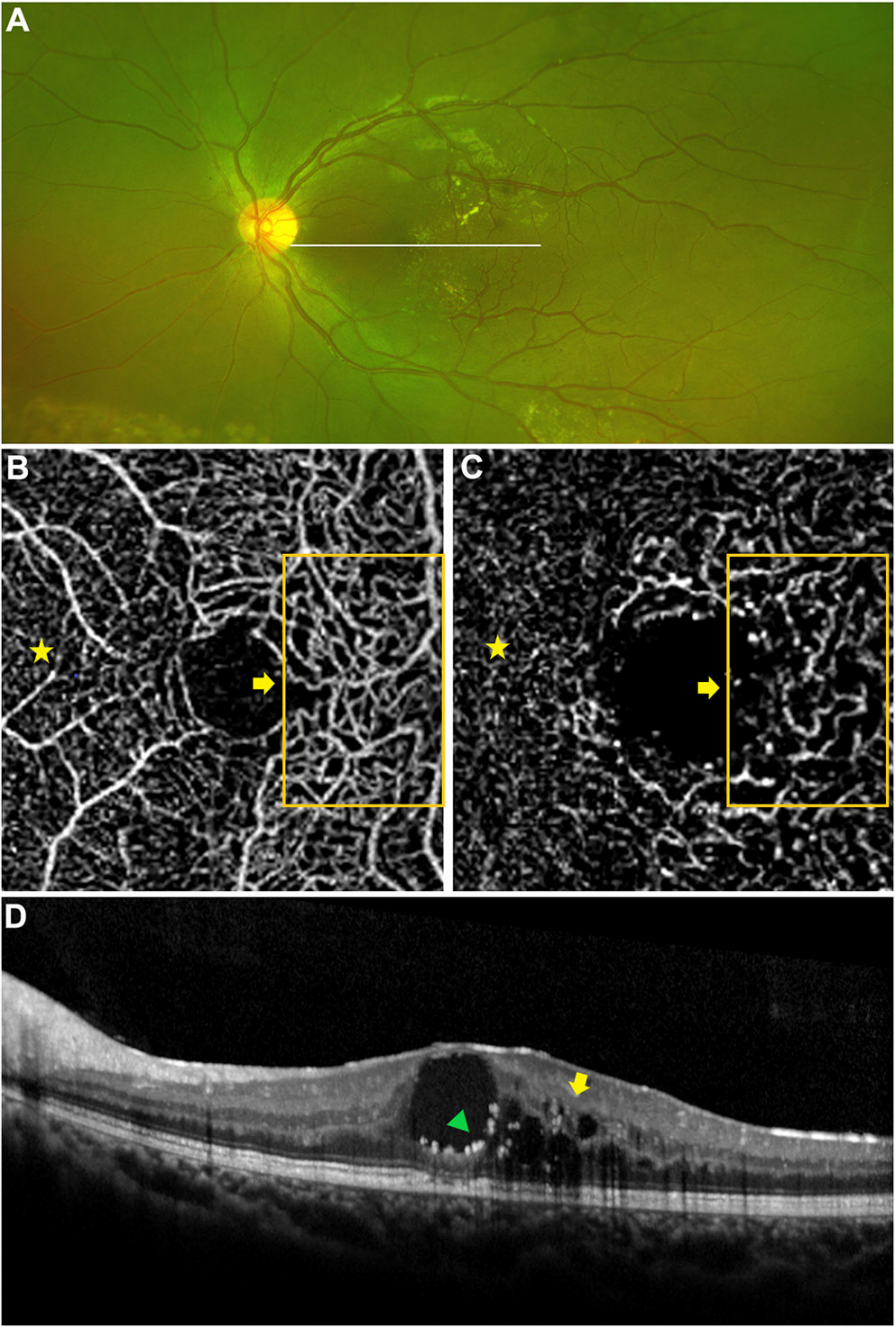
Macular capillary abnormalities in eye with macular edema. A 9 years old boy with Coats' disease presented as concomitant macular edema (edema combined with exudation). Yellow exudates in the macular region on widefield fundus photography **(A)** and intraretinal hyporeflective intraretinal cystoid spaces (yellow arrow) combined with intraretinal hyperreflective exudates (green triangle) on OCT imaging **(D)**. Telangiectasia in the SCP and DCP were detected via OCTA [**(B,C)** 3 × 3 mm], these abnormal capillaries were located in the temporal region of the macula [**(B,C)** orange boxes and yellow arrows], which presented as more dilated microvascular structures in contrast to the capillaries in the nasal region of the macula (yellow stars), in correspondence to the location of macular lesion on OCT imaging [**(D)** yellow arrow, green triangle].

**Figure 3:**
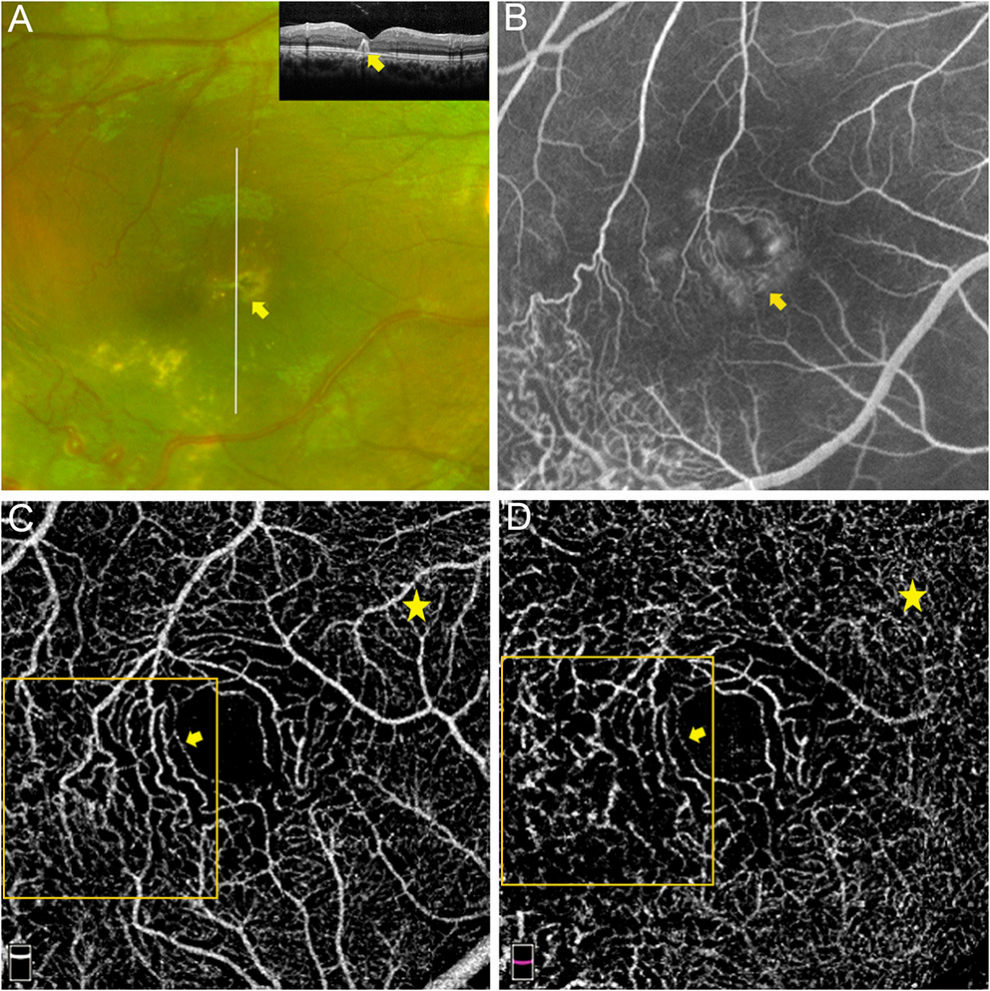
Macular capillary abnormalities in eye with macular exudation. A 8 years old boy with Coats' disease presented as intraretinal exudation in the macular region. Yellow exudates in the macular region on widefield fundus photography and hyperreflective exudates involving the neuro-retina on OCT imaging [**(A)** yellow arrows]. Hyperfluorescent leakage and vague telangiectasia in the macular area on FA [**(B)** captured at 14.3 s]. Telangiectasia and capillary tortuosity in the SCP [**(C)** 3 × 3 mm] and DCP [**(D)** 3 × 3 mm] were detected via OCTA (orange boxes and yellow arrows), which presented as more dilated and tortuous microvascular structures in contrast to the capillaries in the nasal region of the macula (yellow stars).

The morphological abnormalities of microvasculature in the macular region presented as telangiectasia and capillary tortuosity, which were defined as obviously more dilated or tortuous microvascular structures in the local region in contrast to the surrounding capillaries in the same OCTA and FFA images (Figures 1C, 2B,C, 3C,D).

All image analyses, except for the automatically quantitative analyses via built-in software, were conducted by two experienced ophthalmologists (Juan Zhang and Lu Ruan) in a masked manner and, respectively at different times for twice to ensure the objectivity of the evaluation. The analysis results of all included images were consistent.

### Follow-Up and Treatment

SD-OCT and widefield fundus photography were performed at each follow-up visit. After a follow-up of 18 months, the outcomes of the macular lesions were classified as either improved or refractory. Refractory lesions were defined as persistent, recurrent, or aggravated macular edema or exudation despite active therapy during the follow-up period. Improved lesions were defined as the resolution of macular edema or exudation compared with the baseline status.

The treatments for Coats' disease were classified into three types: (1) laser photocoagulation (LP) targeting the telangiectasia; (2) intravitreal injection of anti-vascular endothelial growth factor (VEGF) agent (Ranibizumab) combined with LP; and (3) combined pars-plana vitrectomy (PPV) for epiretinal membrane and associated complications or retinal detachment with massive subretinal exudates, which did not respond to LP alone.

### Histopathologic Analysis

Paraffin-embedded sections of one additional enucleated eye with stage 5 Coats' disease were obtained from the pathology center at the Eye and ENT hospital. This eye was from a 8-year-old boy with a disease history that his parents found his pupil of this affected eye was white for 3 months, and this affected eye was examined and diagnosed at Eye and ENT Hospital of Fudan University for stage 5 Coats' disease with a total exudative retinal detachment to the posterior of lens and combined neovascular glaucoma, which met the criteria of enucleation therapy. His parents agreed to conduct this therapy and signed the informed consent. The sections were stained with hematoxylin and eosin (HE) and scanned under a light microscope (Leica Microsystems, Bensheim, Germany). A section containing the optic nerve and macula was selected for analysis. Scans were obtained at low- and high-power magnification to determine the existence and classification of telangiectasia and inflammatory cells in the macular region.

### Statistical Analyses

All images were analyzed by three experienced ophthalmologists independently. All data analyses were conducted using SPSS Statistics software version 20.0 (IBM Corp, Armonk, NY). Continuous variables are expressed as medians ± standard deviation (SD). Fisher's exact test was used to compare the gender and clinical stage between the two subgroups. The normal distributions of continuous variables were evaluated by the Shapiro-Wilk test, continuous variables with normal distribution were analyzed using parametric tests as followings. Paired *t-*tests were used to compare continuous variables between the affected eyes and unaffected contralateral eyes. Unpaired *t-*tests were used to compare continuous variables between subgroups of affected eyes. Correlations between the structural variables (VD and FD) were conducted using Pearson's correlation test. Correlations between the presence of vascular abnormalities and subgroups were conducted using Pearson's χ^2^ test. The statistical significance level was set at a *P-*value of <0.05.

## Results

### Patient Characteristics

Twenty-six patients with Coats' disease who were followed up for 18 months were eligible for this study. According to the presence of macular edema, 50.0% of the patients presented with simple macular edema or edema combined with exudation (concomitant macular edema) and 50.0% presented with simple macular exudation. The demographic characteristics of the patients are displayed in Table 1. The mean ± SD BCVA (logMAR) of patients with or without macular edema were 1.40 ± 1.01 and 0.92 ± 0.83, respectively, at baseline (*P* = 0.19), and 1.31 ± 1.10 and 0.87 ±0.77, respectively, at 18 months (*P* = 0.57). The ages, genders, and clinical stages of the patients were comparable in both groups (all *P* > 0.05).

**Table 1:** Demographic characteristics of eyes with Coats' disease with macular edema or exudation.

	**Simple or concomitant macular edema**	**Simple macular exudation**	**All**	***P***
Number of eyes (%)	13 (50.0%)	13 (50.0%)	26 (100.0%)	/
Mean age ± SD, years	12.62 ± 4.44	12.53 ± 3.79	12.57 ± 4.05	0.96^†^
Gender, males / females	12 / 1	12 / 1	26 / 2	1.00^*^
BCVA at baseline, logMAR, mean ± SD	1.40 ± 1.01	0.92 ± 0.83	1.16 ± 0.93	0.19^†^
**BCVA at follow-up for 18M, logMAR, mean ±SD**	1.31 ± 1.10	0.87 ± 0.77	1.10 ± 0.97	0.25^†^
**Stage No. (%)**				0.67^*^
2B	8	10	18	
3A	5	3	8	

*No, number; BCVA, best corrected visual acuity*.

*Statistics: P value: comparisons between the simple or concomitant macular edema group and simple macular exudation group*.

† *Unpaired t-test*.

* *Fisher's exact test*.

### Baseline

#### Morphological Analysis

Among the 26 eyes with macular edema or exudation, OCTA revealed macular microvascular abnormalities, presenting as telangiectasia or capillary tortuosity, in the SCP in nine eyes (34.6%) and in the DCP in 20 eyes (76.9%; Figure 2). FFA revealed telangiectasia in the macular region in just 3 of 14 eyes (21.4%, Figure 3), by contrast, the detection rates in the SCP and DCP of these 14 eyes conducted with FFA were 35.7% (5 eyes) and 85.7% (12 eyes), respectively. The associations between the presence of dilated and tortuous capillaries with the characteristics of macular edema or exudation are summarized in Table 2. The rate of macular capillary abnormalities in the SCP was significantly greater in eyes with macular edema (simple and concomitant) compared with eyes with simple macular exudation (61.5 vs. 7.7%; χ^2^ = 8.327, *P* = 0.004). The rate of macular capillary abnormalities in the DCP was also greater in eyes with macular edema (simple and concomitant) than in eyes with simple macular exudation, although the difference was not statistically significant (92.3% vs. 61.5%; χ^2^ = 3.469, *P* = 0.063). The rate of macular capillary abnormalities in the SCP and DCP in eyes with intraretinal lesions were 57.1 and 92.9%, respectively; these values were significantly greater than those in eyes with subretinal lesions only in the SCP (8.3%, χ^2^ = 6.801, *P* = 0.009) or DCP (58.3%, χ^2^ = 4.338, *P* = 0.037).

**Table 2:** The morphological abnormalities of macular microvasculature in eyes with macular edema or exudation secondary to Coats' disease.

	**Presence of macular microvascular abnormalities on OCTA**	
	**Existence in SCP, No. (%)**	**Existence in DCP, No. (%)**
**Existence of macular edema**		
Yes	8/13 (61.5%)	12/13 (92.3%)
No (simple macular exudation)	1/13 (7.7%)	8/13 (61.5%)
χ^2^	8.327	3.469
*P* value	**0.004**	0.063
**Location of macular lesions**		
Intraretinal	8/14 (57.1%)	13/14 (92.9%)
Subretinal	1/12 (8.3%)	7/12 (58.3%)
χ^2^	6.801	4.338
*P*-value	**0.009**	**0.037**

*No, number; SCP, superficial capillary plexus; DCP, deep capillary plexus*.

*Macular lesions (cystoid or exudates) were divided as intraretinal lesions and subretinal lesions respectively, intraretinal lesions in the macular region were defined as cystoid or exudates involving the neuroretina, whereas subretinal lesions were defined as exudates or fluid located only under the neuroretina*.

*Statistics: Pearson's χ2 test. Values in bold font are statistically significant at P < 0.05*.

#### Quantitative Analysis

**Figure 4:**
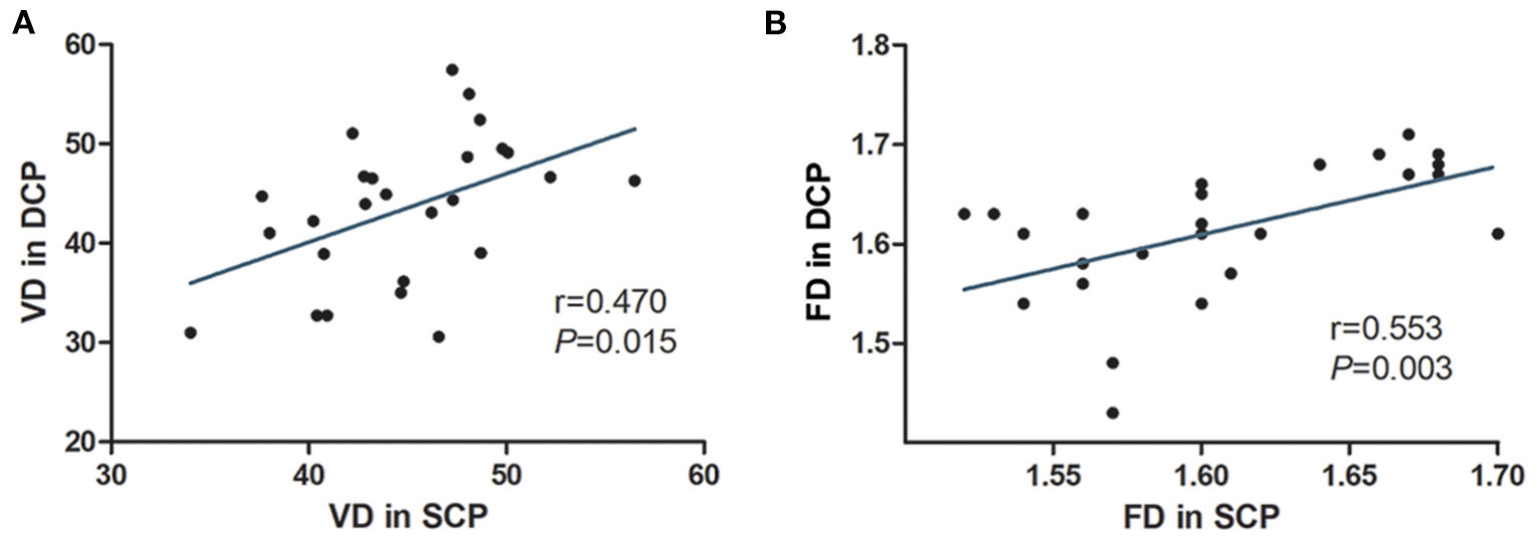
The correlations of OCTA parameters of eyes with macular edema or exudation secondary to Coats' disease. The parafoveal VD in the SCP of the affected eyes was linearly correlated with that in the DCP **(A)**. The parafoveal FD in the SCP of the affected eyes was linearly correlated with thast in the DCP **(B)**.

Table 3 shows the macular vascular parameters of these eyes detected quantitatively via OCTA. The contralateral eyes were used as a control group because no vascular abnormalities or exudates were detected by wide-field photography in those eyes. The parafoveal VDs of the SCP and DCP were significantly lower in the affected eyes than in the contralateral eyes (both *P* < 0.001, Table 3). The FD of the SCP and DCP were also significantly lower in the affected eyes (both *P* < 0.001). The parafoveal VDs of the SCP and DCP were positive correlated (*r* = 0.470, *P* = 0.015). The parafoveal FD of the SCP and DCP were also positively correlated (*r* = 0.553, *P* = 0.003) (Figure 4).

**Table 3:** OCTA parameters of eyes with macular edema or exudation secondary to Coats' disease.

	**Contralateral Eyes**	**Affected eyes**				P*
		**ALL**	**Stage 2B**	**Stage 3A**	***P***	
**Vessel density (%)**						
SCP (SD)	53.29 ± 4.06	44.84 ± 4.97	45.89 ± 4.75	41.31 ± 4.29	**0.045**	**<0.001**
DCP (SD)	59.67 ± 3.49	43.45 ± 7.29	45.08 ± 6.60	38.01 ± 7.40	**0.035**	**<0.001**
**Fractal dimension**						
SCP (SD)	1.659 ± 0.038	1.605 ± 0.052	1.61 ± 0.06	1.58 ± 0.03	0.196	**<0.001**
DCP (SD)	1.659 ± 0.039	1.612 ± 0.053	1.62 ± 0.06	1.57 ± 0.07	0.081	**<0.001**
**Retinal thickness**						
Foveal whole layer, μm	219.38 ± 25.53	332.19 ± 161.81	320.93 ± 161.09	369.75 ± 173.47	0.556	**0.001**
Parafoveal whole layer, μm	331.87 ±21.59	410.59 ± 75.98	406.20 ± 77.09	425.26 ± 77.14	0.609	**<0.001**
AL, mm	22.93 ± 0.76	22.57 ± 0.83	22.63 ± 0.87	22.34 ± 0.66	0.468	0.108

*SCP, superficial capillary plexus; DCP, deep capillary plexus; SD, standard deviation; AL, axial length*.

Statistics: P^*^ value: comparisons between affected eyes and contralateral eyes, Paired t-test. P value: comparisons between affected eyes in stage 2B and stage 3A, Unpaired t-test. Values in bold font are statistically significant at P < 0.05.

We also divided the affected eyes into two subgroups based on the clinical stage. The VDs of the SCP and DCP were significantly lower in eyes classified as stage 3A (*n* = 6) compared with eyes classified as stage 2B (*n* = 20; *P* = 0.045 and *P* = 0.035, respectively). The FDs of the capillary layer and the retinal thicknesses did not differ significantly between the stage 3A and stage 2B eyes (all *P* > 0.05). The ALs were comparable among each subgroup (all *P* > 0.05).

### Follow-Up for 18 Months

**Figure 5:**
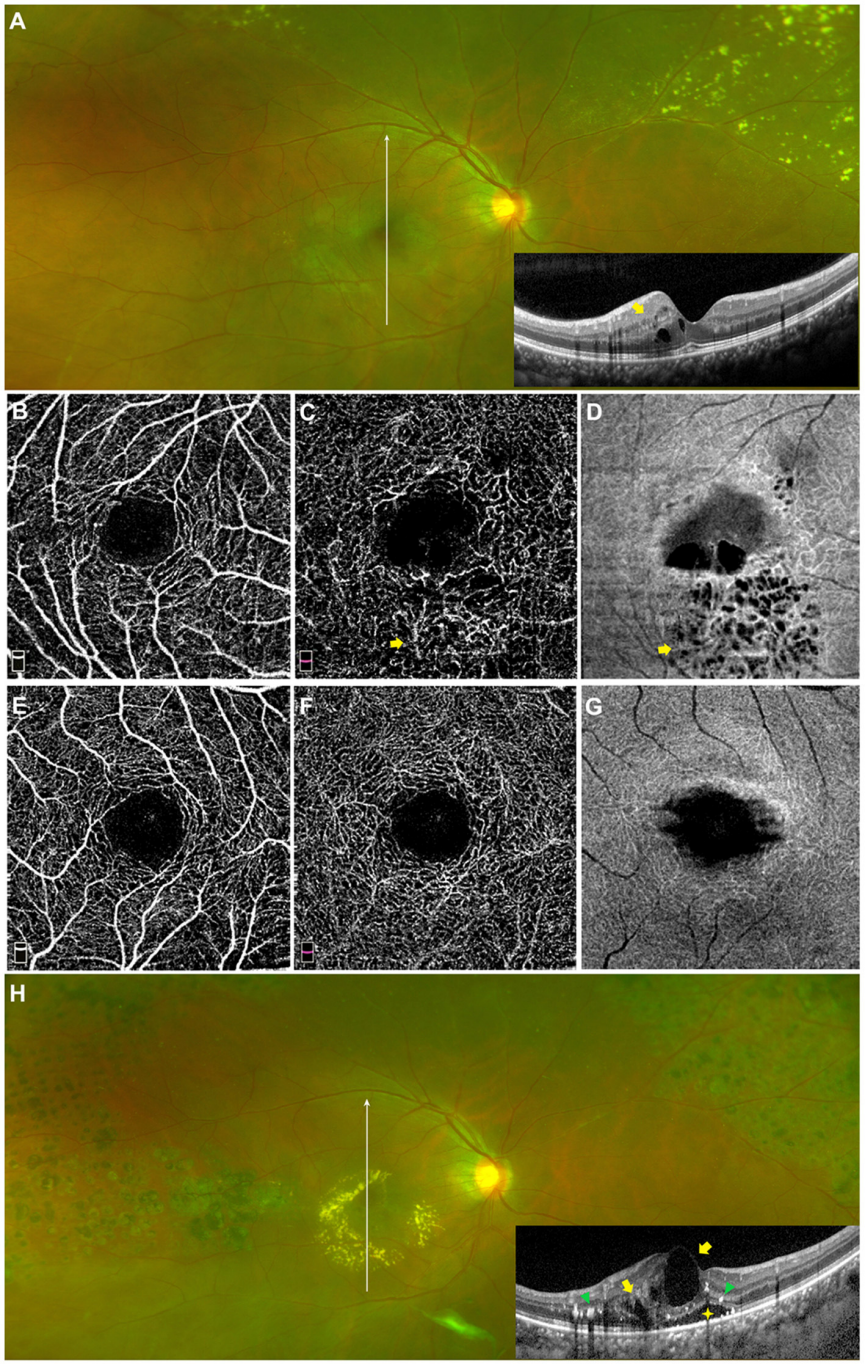
Macular capillary loss at baseline in eye with refractory macular edema. A 17 years old juvenile with Coats' disease presented as refractory macular edema. Macular edema on widefield fundus photography and on OCT imaging at baseline [**(A)** yellow arrow]. Parafoveal VD losses in the SCP [**(B)** VD: 38.5%] and DCP [**(C)** VD: 41.2%] of the affected eye at the baseline visit were quantitatively detected via OCTA (3 × 3 mm), by contrast, parafoveal VDs in the SCP **(E)** and DCP **(F)** of the contralateral eye were 54.1 and 61.3%, respectively. Telangiectasia in the DCP of the affected eye at baseline was also revealed, which was located in the parafoveal inferior region on OCTA enface imaging [**(C)** yellow arrow], in correspondence to the hyporeflective cystoids on OCT enface imaging [**(D)** yellow arrow]. OCT enface imaging targeting on the macula of the contralateral eye **(G)**. The macular lesion in the affected eye aggravated and presented as enlarged intraretinal cystoid spaces (yellow arrows) with hyperreflective exudates (green triangles) as well as subretinal exudates and fluid (yellow star) **(H)**.

Twenty-six affected eyes enrolled in this study were all followed for 18 months and classified according to the clinical outcome of macular edema or exudation, and the associations between the macular microvascular losses at the baseline visit and the clinical outcomes are shown in Table 4. The VDs of the SCP and DCP were both significantly lower in the refractory group than those in the improved group (*P* = 0.009 and *P* = 0.010, respectively), but the FDs of the SCP and DCP were not significantly different between the two groups (both *P* > 0.05). The AL variables in these two groups were comparable (*P* = 0.740), the treatments that patients received in these two groups were also comparable (*P* = 0.763), in the improved group, 5 (45.4%) patients received LP depending on their existence of peripheral dilated capillaries, 3 (27.3%) patients received intravitreal injection of Ranibizumab for 3 times and 3 (27.3%) patients received a combined PPV; in the refractory group, 5 (33.3%) patients received LP depending on their existence of peripheral dilated capillaries, 4 (26.7%) patients received intravitreal injection of Ranibizumab for three times and 6 (40.0%) patients received a combined PPV. Figure 5 shows an affected eye with macular telangiectasia and VD loss at the baseline visit that progressed to refractory macular edema, the macular lesion aggravated and presented as enlarged intraretinal cystoid spaces with hyperreflective exudates as well as subretinal exudates and fluid, despite the peripheral dilated capillaries were all ablated by laser photocoagulation.

**Table 4:** The association of macular vascular variables at baseline with the intractable nature of macular lesions.

	**Improved**	**Refractory**	***P-*value**
**Vascular density (%)**			
SCP	47.69 ± 4.41	42.73 ± 4.36	0.009^*^
DCP	47.60 ± 6.69	40.39 ± 6.29	0.010^*^
**Fractal dimension**			
SCP	1.62 ± 0.05	1.60 ± 0.05	0.629^*^
DCP	1.62 ± 0.06	1.61 ± 0.07	0.604^*^
**Treatment**			0.763^‡^
LP	5 (45.4%)	5 (33.3%)	/
LP + IVR	3 (27.3%)	4 (26.7%)	/
Combined PPV	3 (27.3%)	6 (40.0%)	/
AL, mm	22.63 ± 0.86	22.51 ± 0.83	0.740

*SCP, superficial capillary plexus; DCP, deep capillary plexus; LP, laser photocoagulation; IVR, intravitreal injection of Ranibizumab; PPV, pars-plana vitrectomy; AL, axial length*.

*Statistics*:

* *Unpaired t-test*.

‡ *Fisher's exact test. Values in bold font are statistically significant at P < 0.05*.

### Histopathological Analysis

**Figure 6:**
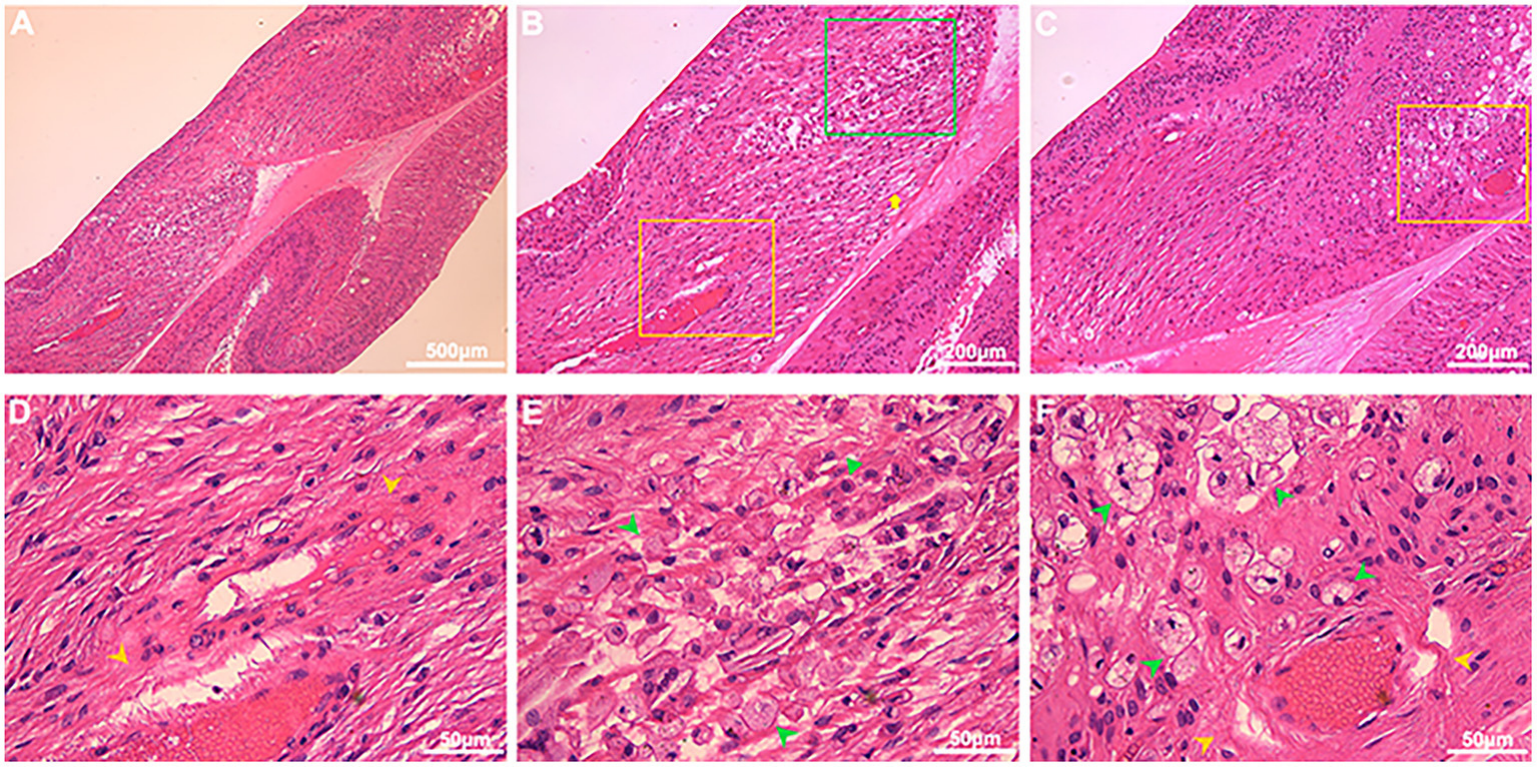
Histopathological features in the macular region of Coats' disease. HE staining from an enucleated eye of Coats' disease in stage 5, which presented as whole retinal detachment. On low-power light micrographs [**(A)** 5×; **(B,C)** 10×], dilated capillaries in the macular region (yellow boxes) were observed, and the parafoveal tissue architecture were also disrupted and replaced by hyperplastic collagen fibers (yellow arrow), infiltrated inflammatory cells and vacuoles [**(B)** green box; **(C)** yellow boxes]. The yellow boxes in **(B,C)** denoting the areas with dilated capillaries were shown in the high-power micrographs [**(D,F)**, respectively; 40×], the green box denoting the area with inflammatory cells and vacuoles was shown in the high-power micrograph [**(E)** 40×], the vessel walls of dilated capillaries were incomplete [**(D,E)** yellow arrowheads], pigment-containing macrophages (green arrowheads) and plasma cells (green triangle) were located adjacent to the dilated capillaries **(E,F)**.

On histologic sectioning with low-power magnification, dilated capillaries in the macular region were observed, and the parafoveal tissue architecture were also disrupted and replaced by hyperplastic collagen fibers, infiltrated inflammatory cells and vacuoles (Figures 6A–C). On the high-power magnification, the vessel walls of dilated capillaries were incomplete (Figures 6D,F). Pigment-containing macrophages and plasma cells were located adjacent to the dilated capillaries (Figures 6E,F).

## Discussion

The origin of macular edema or exudation and whether these defects are due to macular microvascular abnormalities are unclear. In the present study, we enrolled a relatively large sample of children with Coats' disease and combined multimodal ophthalmological images with histopathological analysis to detect macular capillary abnormalities in eyes with macular edema or exudation morphologically and quantitatively. To the best of our knowledge and literature searches, this is the first cross-sectional and longitudinal study to analyze the correlation between the features and progression of macular edema or exudation in eyes with Coats' disease and macular capillary abnormalities.

Previous researchers hypothesized that macular exudation could be caused by peripheral retinal vessel abnormalities based on the discrepancy between the high prevalence of macular exudation and the rarity of macular telangiectasia on color photography, and the concurrence of macular exudation and abnormal peripheral retinal vessels (2–4, 8). However, because FFA is limited in its ability to perform retinal segmentation, the macular vessel abnormalities are often unclear or of low detail. In our study, OCTA revealed the presence of dilated and tortuous capillaries in the macular region, especially in the DCP, in eyes with macular edema or exudation associated with Coats' disease, with detection rates (both for 14 eyes conducted with FFA and all 26 eyes) much higher than that determined by FFA. Considering the limited ability of FFA to visualize the microvasculature in the DCP, this difference suggests the high probability of macular capillary abnormalities in eyes with Coats' disease and that the previous understanding of macular capillary abnormalities and their correlations with macular edema or exudation may be subject to technique limitations. It is also worth noting that the rate of telangiectasia in the DCP of affected eyes was higher than that in the SCP. Primate studies have demonstrated that the DCP sprouts from the SCP and is a terminal microvasculature with weaker vessel walls (18, 19). Thus, our findings may indicate that the weaker distal capillaries of the macula may be more likely to be disrupted in Coats' disease.

In our correlation analyses, we found that eyes with macular edema (simple and concomitant) were more likely to have dilated and tortuous capillaries in the SCP and DCP in the macular region than eyes with simple macular exudation, although only the rate of abnormal capillaries in the SCP was significantly different between these two subgroups. Besides, eyes with intraretinal hyporeflective cystoid spaces or hyperreflective exudates in the macular region were significantly more combined with macular dilated and tortuous capillaries in both SCP and DCP than eyes only with subretinal exudates or fluid. These findings indicate that intraretinal exudates and hyporeflective cystoid spaces in the macula region may be closely related to the macular dilated and tortuous capillaries. The histopathologic sections provide support to this suggestion, we observed that dilated capillaries with incomplete vessel walls also existed in the macular region. We also observed inflammatory cells adjacent to these abnormal capillaries accumulated and disrupted the normal intraretinal tissue architecture resulting in vacuoles and clumps of inflammatory cells, which may be correspond to the macular edema and exudation in the retina depicted by OCTA. Overall, these findings suggest that the intraretinal cystoid spaces and exudates in the macula secondary to Coats' disease may result from the macular abnormal capillaries. As far as we know, this histopathological analysis of microvasculature defects and their possible relationship with inflammatory cells was firstly reported in the macular region in this study.

Shields et al. described retinal capillary dropout adjacent to telangiectasia in the peripheral regions on fundus FFA images, (2) but the existence of macular capillary dropout was unknown. More recently, Brockmann et al. reported an enlarged foveal avascular zone in eyes with stage 1–2 Coats' disease (19 eyes) compared with the contralateral eyes, but the difference was not significant (12). Schwartz et al. reported that the parafoveal VD was decreased in eyes with Coats' disease (13 eyes) compared with unaffected contralateral eyes, (13) but the sample was relatively small and the clinical significance of the decreased VD was not determined.

In our study, we sought to confirm the existence of parafoveal VD loss in eyes affected by Coats' disease and determine its clinical significance, particularly its relationship with macular lesions. To achieve this, we enrolled a relatively large number of patients with eyes displaying macular edema or exudation secondary to Coats' disease and followed them for over 18 months. Considering that widefield imaging revealed abnormal peripheral vessels in the unaffected eyes of patients with Coats' disease in prior studies (8, 12), widefield imaging of the unaffected eyes was performed in our study at baseline and during the follow-up period to ensure they did not have abnormal vessels or exudation. This allowed us to use the unaffected eyes as a control group. First, we found significant losses of the parafoveal VDs of the SCP and DCP in affected eyes compared with contralateral eyes. The parafoveal VDs of both layers tended to decline with increasing stage of Coats' disease. Furthermore, the FDs, which have been used to evaluate the loss of smaller diameter vessels and the microvascular architecture (17, 20), were also lower in both layers in affected eyes than in contralateral eyes, although they were not significantly different between stages 2B and 3A. These findings indicate the possibility of macular capillary dropout in eyes with macular edema or exudation associated with Coats' disease and these parameters may reflect the severity of Coats' disease. In addition, we found that VD/FD in the SCP was significantly correlated with that in the DCP, indicating that the loss of capillaries in SCP is consistent with that in the DCP. These findings could be explained by the retinal vascular anatomy and the developmental pattern aforementioned (18, 19).

Refractory macular edema or exudation is a challenge to the clinical management of Coats' disease. However, few studies have examined the causes and associated risk factors, which remain largely unknown. In consideration of this background, we divided the affected eyes according to the outcomes of the macular lesions during the follow-up (i.e., improved or refractory). As illustrated in the example shown in Figure 5, eyes with macular edema or exudation, which progressed to persistent, recurrent, or aggravated macular edema or exudation despite resolution of peripheral telangiectasia and exudates by active therapy, presented with dilated and sparse capillaries in the macular region at baseline. Quantitative analysis, under the condition of comparable treatments in each subgroup, revealed significantly worse parafoveal VD loss in the SCP and DCP at baseline in the refractory group than the improved group. FD was mainly used to be evaluate the loss of smaller diameter vessels, the FD in SCP and DCP were lower in the refractory group than in the improved group, but the differences were not statistically significant. These results suggest that the sensitivity of FD may be inadequate in eyes with telangiectasia combined with VD loss.

The findings described above indicate that parafoveal VD losses in the SCP and DCP at baseline may be risk factors for refractory macular edema or exudation in eyes with Coats' disease. Similarly, VD loss and macular ischemia were reported to be risk factors for refractory macular edema secondary to branch retinal vein occlusion in eyes with poor responses to anti-VEGF agents (21, 22). Some studies revealed that anti-VEGF agents reduced the normal vessel density (23, 24). Therefore, we speculate that excessive anti-VEGF therapy may aggravate refractory macular edema in eyes with Coats' disease by reducing the parafoveal VD. Accordingly, the treatment strategies for refractory macular edema or exudation need to be updated. Studies examining the longitudinal changes in parafoveal VD (and its loss) at each follow-up are necessary to provide more evidence for this speculation.

Taken together, the findings above provide important evidence about the roles of macular capillary abnormalities in the pathology of Coats' disease. Coats' disease is currently staged according to the classification proposed by Shields et al., and substages are defined basing on the presence of extrafoveal (A) or foveal (B) exudates (25) We recommend that the presence of macular capillary abnormalities should be considered as a supplement to the current clinical staging system.

We are aware that our study has several limitations. Although our study comprised the largest number of patients with macular edema or exudation associated with Coats' disease who underwent OCTA to date, it was still relatively small due to the rarity of Coats' disease, especially those presenting with macular edema or exudation without macular fibrosis and a history of previous therapy. The OCTA variables were automatically calculated using the built-in software, but image artifacts and segmentation errors were manually excluded, and the comparable AL in different groups could help to eliminate the image magnification error due to differences in AL. Besides, FFA was not conducted in all affected eyes and their contralateral eyes because FFA is an invasive method and not safe enough for these pediatric patients of Coats' disease, which might limit the certainty that the contralateral eyes were disease-free. To overcome this limitation, we conducted widefield fundus photography at each follow-up visit for both eyes to exclude any vascular abnormalities in contralateral eyes alternatively. Another limitation may be that the contralateral eyes of Coats' disease were not compared with healthy eyes. Despite these limitations, we believe that our results provide valuable insights into the macular capillary abnormalities in eyes affected by Coats' disease and improve our understanding of the underlying vascular pathology.

In conclusion, we investigated that macular dilated and tortuous capillaries, especially in deep capillary plexus, highly existed in corresponding to macular edema or exudation and were correlated with the formation of intraretinal cystoid spaces and exudates of the macula in Coats' disease. These findings are supported by the histopathological finding, including dilated capillaries with incomplete vessel walls, together with adjacent accumulated inflammatory cells and vacuoles. Furthermore, the parafoveal VDs of the SCP and DCP were lower in eyes with macular edema or exudation associated with Coats' disease and declined with increasing clinical stage. Parafoveal VD losses in the SCP and DCP at baseline may be a risk factor for refractory macular edema or exudation secondary to Coats' disease.

## Data Availability Statement

The raw data supporting the conclusions of this article will be made available by the authors, with undue reservation.

## Ethics Statement

Ethical review and approval was not required for the study on human participants in accordance with the local legislation and institutional requirements. Written informed consent to participate in this study was provided by the participants' legal guardian/next of kin.

## Author Contributions

JZ and LR conducted this study, analyzed images, conducted statistical analysis, and wrote the manuscript. CJ, QY, and YJ examined patients. XH and QC designed this study and revised the manuscript. All authors contributed to the article and approved the submitted version.

## Funding

This study was supported by research grants from the National Science Foundation of China (82070975) and the Health Science and Technology Project of the Pudong New District Commission of Health and Family Planning, Shanghai (PW2016D-12).

## Conflict of Interest

The authors declare that the research was conducted in the absence of any commercial or financial relationships that could be construed as a potential conflict of interest.

## Publisher's Note

All claims expressed in this article are solely those of the authors and do not necessarily represent those of their affiliated organizations, or those of the publisher, the editors and the reviewers. Any product that may be evaluated in this article, or claim that may be made by its manufacturer, is not guaranteed or endorsed by the publisher.
